# A Cross-Sectional Study on the Relationship Among Cytokines, 5-HT2A Receptor Polymorphisms, and Sleep Quality of Non-manual Workers in Xinjiang, China

**DOI:** 10.3389/fpsyt.2022.777566

**Published:** 2022-04-06

**Authors:** Juan Wang, Xiaoyan Gao, Pengcheng Gao, Jiwen Liu

**Affiliations:** Department of Public Health, Xinjiang Medical University, Urumqi, China

**Keywords:** *5-HTR2A*, cytokines, sleep quality, non-manual workers, cross-sectional study

## Abstract

**Background:**

Studies have shown that cytokine activity changes during the sleep-wake process, suggesting that inflammatory factors may be involved in a mechanism affecting sleep quality. Furthermore, the serotonergic system is also one of the essential components of airway relaxation during sleep, especially the serotonin 2A receptor (5-HTR2A) type that plays an important role in the sleep-wake process. Therefore, this research aimed to investigate the effects of cytokines and *5-HTR2A* polymorphisms on sleep quality in non-manual workers in Urumqi, Xinjiang in order to explore the relationship between the three.

**Methods:**

This study used a cluster sampling method to randomly select non-manual workers who worked in Urumqi, Xinjiang for at least 1 year. From July 2016 and December 2017, this study recruited 1,500 non-manual workers for physical examination in the First Affiliated Hospital of Xinjiang Medical University. According to the inclusion and exclusion criteria, 1,329 non-manual workers were finally included in the questionnaire study. It used the Pittsburgh Sleep Quality Index questionnaire to assess sleep quality. Moreover, another 15% of respondents were randomly selected as the experimental study group. The polymerase chain reaction restriction fragment length polymorphism was used to detect *5-HTR2A* gene genotypes. Simultaneously, the cytokine (IL-1β, IL-2, IL-6, and TNF-α) content was evaluated using an enzyme-linked immunoassay.

**Results:**

The results showed that among the 1,329 respondents, 870 had sleep quality problems, and the detection rate was 65.46%. The distribution of −1438G/A genotypes in the *5-HTR2A* gene was significantly different among different sleep quality groups (*p* < 0.05), with no statistical significance present when comparing to T102C (*p* > 0.05). Logistic regression analysis showed that the AG [odds ratio (OR) = 2.771, 95% confidence interval (CI): 1.054–7.287] and GG (OR = 4.037, 95% CI: 1.244–13.105) genotypes at −1438G/A loci were both associated with poor sleep quality and were thus considered the susceptibility genotypes for sleep problems. Furthermore, IL-1β was shown to be a protective factor for sleep quality (OR = 0.949, 95% CI: 0.925–0.974). The interaction results showed that AG × IL-1β (OR = 0.952, 95% CI: 0.918–0.987) was associated with a lower risk of sleep problems than AA × IL-1β.

**Conclusion:**

Cytokines and *5-HTR2A* polymorphisms not only have independent effects on sleep but also may have cumulative effects. Therefore, it is necessary to further explore the related mechanisms affecting sleep quality to improve the sleep quality of non-manual workers.

## Introduction

Sleep is a complex physiological process that accounts for about one-third of human life (1, 2). Good quality sleep can regulate a variety of physical and psychological activities that are beneficial to human health. The term “sleep quality” is commonly used in sleep medicine, yet a standard definition of sleep quality has not been identified. “Sleep quality” is sometimes used to refer to a collection of sleep measures including total sleep time (TST), sleep onset latency (SOL), degree of fragmentation, total wake time, sleep efficiency, and sometimes sleep disruptive events such as spontaneous arousals or apnea (3). Poor sleep quality is a subjective experience in which the amount and/or quality of sleep is insufficient and interferes with daily functioning during the day. The occurrence of sleep problems may cause adverse reactions, such as lethargy and fatigue, increase susceptibility to psychological problems, including anxiety and depression, and elevate the risk of death and cardiovascular diseases like hypertension and coronary heart disease (4–8). Thus, sleep problems have gradually become a common phenomenon, placing a heavy burden on health services worldwide (9).

At present, the incidence of sleep problems varies among different occupational groups in different regions of the world. For example, the incidence rate of insomnia among Japanese workers is 23.2–39.2% (10) and 33.2% in Iranian bank staff (11). The research has shown that manual labor has fixed hours, while white-collar occupations have unlimited hours and often longer than expected (12). There are several studies reporting sleep disturbances in shift workers in manual or service occupations. However, only a few studies have investigated sleep problems in white-collar workers. White-collar workers are described as being associated primarily with higher education and specific skills, or with low-skilled jobs that are primarily social rather than physical (13). Studies have found that long working hours affected sleep quality among male workers and female non-manual workers (14, 15). With the rapid development of the social economy, the form of work gradually changed to mental labor. Non-manual workers, namely white-collar workers, may experience more stress and a fast-paced lifestyle and become prone to anxiety, depression, and other psychological problems, which may affect their sleep quality. Therefore, the sleep problems of non-manual workers cannot be ignored.

Sleep is affected by various aspects. In addition to the environmental factors, such as stress and demands associated with work (16), the impact of physiological and genetic factors on sleep has also been extensively studied. There is evidence of a link between sleep and immune function (17). IL-1β and TNF can accumulate after prolonged wakefulness and appear to promote sleep (18, 19). And cytokines are small-molecule soluble proteins that induce changes in local states by modulating neural activity affecting sleep processes (20). The studies have shown that the expression levels of pro-inflammatory cytokines such as IL-1β and IL-6 are altered with changes in sleep rhythms. Their rising or falling levels can affect nervous system activity, triggering depression and anxiety, which are often accompanied by sleep disorders (21, 22). Studies have found that inflammatory cytokines in the central system are involved in the sleep-wake process. TNF-α can act on established sleep regulation circuits, and its acute enhancement or inhibition can inhibit or promote sleep (23). Furthermore, sleep has long been considered to be regulated by the interplay of circadian and homeostatic mechanisms (24). The immune system modulates circadian rhythms by promoting the expression of immunomodulators that alter sleep rhythm processes mediated by the central nervous system (25, 26). Evidence for the role for IL-1β and TNF in the regulation of physiological sleep has been derived from electrophysiological, biochemical, and molecular genetic studies. IL-1β and TNF increase non-REM (NREM) sleep in several species (rat, mouse, monkey, cat, rabbit, and sheep) irrespective of the route of administration (27, 28).

Among the genetic factors, serotonin (5-HT) can regulate a variety of physiological functions, including appetite, thermoregulation, allodynia, and hormone secretion (29, 30). The observation by Brodie et al. (31) in 1955 that cerebral 5-HT depletion by reserpine induces sedation prompted investigation of the role of 5-HT in the regulation of sleep–wake behavior. The importance of the 5-HT system in sleep regulation is supported by both experimental data and clinical observations: pharmacological manipulations that affect the 5-HT system by altering neurotransmitter synthesis, release, binding, or re-uptake and metabolism result in profound alterations in sleep (18). Its transporter (5-HTT) is an important adjustment factor of the 5-HT activity, which is highly correlated with insomnia (32). The effect of 5-HT is mediated by seven receptor families, includes 5-HT1 to 5-HT7. A variety of studies have demonstrated that 5-HT2 receptors play a major role in the regulation of the sleep-waking cycle. Studies have found that the blockade of 5-HT2 receptors induced significant changes in the phases of sleep and wake in adult rats (33, 34). Ritanserin not only significantly enhanced deep SWS but also improved the index of sleep efficiency (number of intermittent awakenings after sleep onset) in the old rats (35). And ketanserin induced significant differences in the phases of sleep and wake of rats (36). Moreover, the association between the 5-HT2A receptor and sleep has also been found in molecular biology studies. As an important part of the serotonergic system, the expression of the 5-HT2A receptor is controlled by genes. Thus, encoding the receptor polymorphism may affect the functional state of the receptor and thus the activity of 5-HT. Current studies have confirmed that *5-HTR2A* polymorphisms were associated with sleep disorders (37). In addition, the −1438G/A polymorphism of the *5-HTR2A* gene has been found to be associated with the risk of sleep problems in obstructive sleep apnea syndrome (38–42). A genetic association study on the *5-HTR2A* gene revealed a −1438G/A polymorphism, which is a new G-A base change at −1438G/A in the promoter region, which has a very strong linkage imbalance with the T102C polymorphism (43). Therefore, this study mainly evaluated the effects of T102C and −1438G/A in the *5-HTR2A* gene on sleep quality.

Although there have been reports on the effects of cytokines and *5-HTR2A* polymorphisms on sleep, few studies have explored the relationship between cytokines, *5-HTR2A* polymorphisms, and sleep for non-manual workers. Therefore, this study carried out a cross-sectional study in Urumqi, Xinjiang, taking non-manual workers as research objects to investigate and evaluate their sleep quality. The contents of cytokines (IL-1β, IL-2, IL-6, and TNF-α) and the 5-HTR2A polymorphisms were also detected, further examining the independent and interactive effects of cytokines and *5-HTR2A* polymorphisms on sleep quality from the molecular biological perspective. To explore the related mechanism and influencing factors of poor sleep quality, and provide a theoretical basis for improving the sleep status, physical, mental health, and quality of life of non-manual workers.

## Methods

### Subjects

This study was carried out at the Physical Examination Center of the First Affiliated Hospital of Xinjiang Medical University, and the survey period was from July 2016 to December 2017. The questionnaire was conducted in conjunction with the physical examination of the non-manual workers. The study protocol was approved by the Ethics Committee of Xinjiang Medica University, and all participants voluntarily provided their written informed consent before the investigation. The study subjects were non-manual workers working in the Urumqi of Xinjiang Province, China. According to the Occupational Classification Code of the People's Republic of China, the occupational groups of the first and second categories (administrative state organizations, party and mass organizations, enterprises, institutions, professional, and technical personnel) were selected as the overall target. A total of 1,500 non-manual workers (teachers, civil servants, and doctors) were recruited using the cluster sampling method.

The inclusion criteria were as follows: individuals who worked for more than 1 year and were between 20 and 60 years old. The exclusion criteria included the following: history of cardiovascular disease, mental disease, thyroid disease, and other diseases that may cause sleep disorders; sleep apnea syndrome, narcolepsy, restless leg syndrome; and hospitalization or medication for sleep disorders taken within 3 months.

A total of 1,500 questionnaires were sent out, and 1,329 valid questionnaires were recovered after excluding unqualified participants and questionnaires with <80% of the contents. The effective recovery rate of the questionnaires was 88.6%. The sample size was calculated by the formula:


(1) $$\begin{array}{l} \text{n}=\frac{{Z_{\alpha }}^{2}\pi \left(1-\pi \right)}{\delta^{2}} \end{array}$$

where *n*, sample size; *Z*, statistics for significance tests; *Z*_α_ = 1.96 (α = 0.05); π, the prevalence of population; δ, Tolerable error. According to the findings of the Chinese Sleep Association, the prevalence of sleep disorders in China was 38.2% (44). When the allowable error was <3%, the sample size was calculated to be 1,008. However, since cluster sampling was adopted in this study, it should be increased by 50%, and the sample content of this study was finally calculated to be 1,500.

In addition, 200 participants (15% of respondents) were randomly selected as the subjects of the experiment. A total of 166 subjects were tested after excluding individuals that did not meet the criteria for DNA extraction, as well as those with hemolysis and apparent sediment present during the test, which severely affected the cytokine determination results. Then, sex and age were used as matching factors to conduct 1:1 propensity score matching (PSM) on 166 subjects, and 68 pairs were successfully matched for a total number of 136 subjects included in the final analysis.

## Measures

### Sleep Quality

The Pittsburgh Sleep Quality Index (PSQI) (45) was used to evaluate the sleep status of non-manual workers because it was easy to use, has high reliability and validity, and is highly correlated with polyhypnotic electroencephalogram test results. It has become a commonly used scale for clinical evaluation in psychiatric departments abroad. According to the Chinese version of the reliability and validity study as well as the study carried out by Tsai et al. (46), the Chinese version of the PSQI (CPSQI) has shown good internal consistency and reliability (Cronbach's α = 0.82–0.83). And the retest reliability of 14–21 days was 0.85 (all subjects) and 0.779 (primary insomnia) (47). The table was composed of 19 self-rated and five other items (not included in scoring). The scoring items were subjective sleep quality, sleep time, sleep efficiency, sleep disorder, use of hypnotic drugs, and daytime dysfunction. Each component was scored on a scale from 0 to 3, and the total score of each component was the total PSQI score ranging from 0 to 21. The higher the score, the worse the sleep quality. Sleep quality problems were defined using a threshold of 5, with scores ≥5 considered to indicate poor sleep quality (37).

### Blood Sample Collection and Preservation

Venous blood samples were obtained as part of a health examination. High-fat diet and alcohol consumption were avoided for 3 days prior to blood collection. Blood samples were collected between 9 and 11 a.m. by medical staff at the medical center, where 4 mL of elbow venous blood were drawn on an empty stomach, collected in an ethylenediaminetetraacetic acid anticoagulant tube (namely an anticoagulant containing ions) placed in an icebox at −4°C, transported back to the laboratory, and cryopreserved at −20°C until use.

### Cytokine Assay

The IL-1β, IL-2, IL-6, and TNF-α content was assayed using an enzyme-linked immunoassay strictly according to the manufacturer's instructions (Xinze Baoxin Biotech, Urumqi, China) and measured with an automatic microplate analyzer (Model 680; Bio-Rad, Hercules, CA, USA).

### Genotyping

Genomic DNA was extracted using the Whole Blood Genome Extraction kit (Tiangen Biotech, Beijing, China). The polymerase chain reaction-restriction fragment length polymorphism technique was used to analyze polymorphisms. The total volume of each reaction mixture was 20 μL, and gDNA was amplified using PCR instruments (My Cycler; Bio-Rad). A total of 10 μL of PCR product were used in each enzyme-digested mixture, with the final volume totaling 30 μL. The fragments were analyzed using electrophoresis on a 2.5% agarose gel and visualized with ultraviolet light. The primers and genotypes for T102C and −1438G/A are listed in Tables 1, 2.

**Table 1:** PCR primer sequences.

**Genetic loci**	**Primer direction**	**Sequence**	**Amplified fragment length**
T102C	F	5′-TCTGCTACAAGTTCTGGCTT-3′	342 bp
	R	5′-CTGCAGCTTTTTCTCTAGGG-3′	
−1438G/A	F	5′-AGCCAGTTCAATGGTGAT-3′	404 bp
	R	5′-ATGTCATAAGCTGCAAGG-3′	

**Table 2:** Genotype information for T102C and −1438G/A.

**Genetic loci**	**Enzyme-digested fragment length**	**Genotype**
T102C	342 bp	CC
	216, 126 bp	TT
	342, 216, 126 bp	CT
−1438G/A	404 bp	AA
	153, 251 bp	GG
	404, 153, 251 bp	AG

### Quality Control

Investigators with similar training informed the subjects of the purpose and content of the research, so that they could fully understand the significance of the research and to ensure their active cooperation. Two research team members were utilized for data entry and data verification. All laboratory instruments were calibrated to ensure standard operation before experimental work. During the experiment, the contaminated and clean areas were strictly separated. All samples and reagents were properly stored to prevent cross-contamination.

### Statistical Analysis

Epidata 3.0 (The Epidata Association, Odense, Denmark) was used to establish a database for data entry. SAS 9.4 statistical software (SAS Institute Inc., Cary, NC, USA) was used for data analysis. Comparison of sleep quality problem detection rate, Hardy-Weinberg genetic balance of *5-HTR2A* gene, and genotypes among different sleep quality groups was performed using a chi-square test. Cytokine levels (IL-1β, IL-2, IL-6, and TNF-α) were not consistent with a normal distribution and were represented using M(Q25, Q75). Cytokine level comparisons among different sleep quality groups and *5-HTR2A* genotypes were performed using non-parametric tests. Multiple regression analysis was used to investigate the effect between cytokines and *5-HTR2A* polymorphisms on sleep quality. The PSM method was used for data matching, where the matching error was 0.03. The significance level was set at 0.05 (bilateral).

## Results

### Demographics and Sleep Quality Distribution

Among 1,329 subjects, 870 had sleep quality problems (65.46% detection rate; Table 3). Associations between poor sleep quality and education level, length of service, and monthly income were statistically significant (*p* < 0.05). No significant relationship was identified between poor sleep quality and other variables (*p* > 0.05). Subjects with a bachelor's degree or greater (66.78%) were more likely to have sleep problems than those with a technical secondary school education and lower (55.03%). Subjects whose length of service was 15–30 years (71.37%) were more susceptible to having poor-quality sleep than those with service length of <15 years (63.42%) and >30 years (61.02%). Compared to individuals with a monthly income of ≤3,000 yuan (62.78%), those earning >3,000 yuan (70.21%) had a higher probability of experiencing poor sleep.

**Table 3:** Sleep quality distribution among different demographic characteristics.

**Characteristics**	**Number**	**Poor sleep quality (%)**	***χ* ^2^**	***p-*value**
**Sex**				
Male	516	347 (67.25%)	1.189	0.276
Female	813	523 (64.33%)		
**Age (years old)**				
≤35	589	375 (63.67%)	2.508	0.285
35~50	607	411 (67.71%)		
≥50	133	84 (63.16%)		
**Education**				
Technical secondary school and below	149	82 (55.03%)	8.073	0.004^*^
Bachelor degree or above	1,180	788 (66.78%)		
**Marriage**				
Unmarried	226	150 (66.37%)	0.100	0.752
Married	1,103	720 (65.28%)		
**Job**				
Teacher	811	544 (67.08%)	2.493	0.287
Civil servants	204	130 (63.73%)		
Doctor	314	196 (62.42%)		
**Length of service (years)**				
<15	503	319 (63.42%)	11.171	0.004^*^
15~30	454	324 (71.37%)		
>30	372	227 (61.02%)		
**Professional title**				
Elementary	338	211 (62.43%)	2.568	0.277
Intermediate	639	431 (67.45%)		
Advanced	352	228 (64.77%)		
**Monthly income (yuan)**				
≤3,000	849	533 (62.78%)	7.484	0.006^*^
>3,000	480	337 (70.21%)		

* *p < 0.05*.

### Relationship Between Cytokines and Sleep Quality

The IL-1β content in different sleep quality groups was different (*p* < 0.05), suggesting that cytokines may be involved in sleep regulation (Table 4). However, there was no statistical significance between sleep quality and other cytokines (*p* > 0.05).

**Table 4:** Cytokine comparison between different sleep quality groups.

**Sleep quality**	***n***	**IL-1β**	**IL-2**	**IL-6**	**TNF-α**	**Total**
Non-poor sleep quality	68	29.38 (16.56, 45.94)	40.10 (33.63, 49.20)	64.67 (62.00, 68.35)	51.50 (27.50, 72.75)	136
Poor sleep quality	68	16.88 (10.00, 32.19)	44.10 (33.23, 52.38)	64.00 (60.75, 67.83)	50.25 (28.75, 72.00)	
*Z*		−3.916	−0.494	−1.097	−0.194	
*p-*value		<0.001^*^	0.621	0.273	0.846	

* *p < 0.05*.

### Hardy-Weinberg Genetic Equilibrium Test

Hardy-Weinberg genetic balance test was used to analyze the distribution of T102C and −1438G/A genotypes in the *5-HTR2A* gene. The results showed that the actual values of each genotype were in good agreement with the expected values, and these differences were not statistically significant (*p* > 0.05), which was in agreement with the law of genetic balance (Table 5).

**Table 5:** Hardy-Weinberg genetic equilibrium test.

**Genetic loci**	**Genotype**	**Actual value**	**Expected value**	***N***	**χ^2^**	***p-*value**
T102C	CC	26	33	136	5.767	0.056
	CT	82	68			
	TT	28	35			
−1438G/A	AA	31	35	136	1.451	0.484
	AG	75	68			
	GG	30	33			

### Relationship Between *5-HTR2A* Gene and Sleep Quality

Chi-square test was used to analyze the distribution of T102C and −1438G/A genotypes across different sleep quality groups (Table 6). The results showed that −1438G/A genotypes were different in different sleep quality groups (*p* < 0.05), while T102C genotypes were not statistically significantly different (*p* > 0.05).

**Table 6:** Distribution of T102C and −1438G/A genotypes across different sleep quality groups.

**Genetic loci**	**Genotype**	**Number**	**Poor sleep quality**	**Non-poor sleep quality**	**Total**	**χ^2^**	***P***
T102C	CC	26	10	16	136	2.670	0.263
	CT	82	41	41			
	TT	28	17	11			
−1438G/A	AA	31	9	22	136	7.305	0.026^*^
	AG	75	41	34			
	GG	30	18	12			

* *p < 0.05*.

### Relationship Between Cytokines and *5-HTR2A* Gene

There were no statistically significant differences in cytokine comparisons in different T102C and −1438G/A genotypes (*p* > 0.05; Table 7).

**Table 7:** Distribution of T102C and −1438G/A genotypes across different sleep quality groups.

**Genotype**		**Number**	**IL-1β**	**IL-2**	**IL-6**	**TNF-α**	**Total**
T102C	CC	26	21.88 (10.00, 35.31)	44.10 (34.38, 47.83)	64.00 (61.33, 68.67)	49.00 (23.95, 70.00)	136
	CT	82	23.75 (12.50, 35.50)	42.60 (33.60, 51.93)	64.83 (61.92, 68.09)	57.50 (28.25, 77.63)	
	TT	28	23.12 (11.25, 35.94)	40.65 (31.30, 51.73)	63.67 (61.50, 65.92)	44.50 (28.25, 69.00)	
	*H*		0.679	0.575	0.507	1.555	
	*p*-value		0.712	0.750	0.776	0.459	
−1438G/A	AA	31	26.25 (15.00, 35.00)	40.20 (33.70, 47.20)	64.33 (61.67, 68.33)	54.50 (24.50, 62.50)	136
	AG	75	22.50 (12.50, 35.25)	44.10 (34.10, 51.70)	64.33 (61.33, 68.00)	53.50 (27.50, 78.00)	
	GG	30	23.75 (11.25, 36.56)	40.15 (31.50, 52.75)	64.00 (62.50, 66.41)	47.00 (29.75, 73.00)	
	*H*		0.886	1.920	0.001	0.751	
	*p*-value		0.642	0.383	1.000	0.687	

### Correlation Analysis of Cytokines, *5-HTR2A* Polymorphisms, and Sleep Quality in Non-manual Workers

Sleep quality status served as the dependent variable, while T102C and −1438G/A genotypes and cytokines (IL-1β, IL-2, IL-6, and TNF-α) were considered to be independent variables. Multivariate logistic regression analysis was performed. The results showed that −1438G/A was a risk factor for sleep quality after adjusting for confounding factors, such as length of service, education, and monthly income. Compared to AA, both AG [odds ratio (OR) = 2.771, 95% confidence interval (CI): 1.054–7.287] and GG (OR = 4.037, 95% CI: 1.244–13.105) increased the risk of developing sleep problems. However, T102C genotypes were not associated with an increased risk of sleep problems. In addition, IL-1β was a protective factor of sleep quality (OR = 0.958, 95% CI: 0.933–0.983) (Table 8).

**Table 8:** Logistic regression analysis of cytokines, *5-HTR2A* polymorphisms, and sleep quality.

**Variables**	**β**	**SE**	**χ^2^**	***P***	**OR (95% CI)**
**A1438G**					
AA					Ref
AG	1.019	0.493	4.267	0.039^*^	2.771 (1.054–7.287)
GG	1.396	0.601	5.396	0.020^*^	4.037 (1.244–13.105)
IL-1β	−0.052	0.013	15.605	<0.001^*^	0.949 (0.925–0.974)

* *p < 0.05*.

### Interaction Effect Between Cytokines and *5-HTR2A* Polymorphisms on Sleep Quality in Non-manual Workers

To further explore the potential interaction between cytokines and *5-HTR2A* gene in predicting the risk of sleep quality problems, interaction items between cytokines and −1438G/A genotypes were introduced into the logistic regression model. The results showed that there was an interaction between cytokines and −1438G/A, which was still present after adjusting for confounding factors, such as length of service, education, and monthly income (Table 9). Compared to AA × IL-1β, AG × IL-1β (OR = 0.952, 95% CI: 0.918–0.987) was associated with a lower risk of sleep problems (*p* < 0.05).

**Table 9:** Interaction effect between cytokines and *5-HTR2A* polymorphisms on sleep quality.

**Comparison group**	**Reference group**	**OR (95% CI)**	**AOR (95% CI)**
−1438G/A × IL-1β	AA × IL-1β		
AG × IL-1β		0.979 (0.960–0.999)^*^	0.952 (0.918–0.987)^*^
GG × IL-1β		0.991 (0.964–1.019)	0.964 (0.913–1.017)

*OR, odds ratio; CI, confidence interval*;

* *p < 0.05*.

## Discussion

The aim of this study was to investigate and evaluate the sleep quality of non-manual workers in Urumqi, Xinjiang, and to analyze the effects of cytokines and *5-HTR2A* polymorphisms on sleep quality to explore the relationship among the three. The results showed that of the 1,329 study subjects, 870 had sleep problems, and the detection rate was 65.46%. The distribution of sleep problems was different among non-manual workers with different education levels, lengths of service, and monthly incomes. For professionals, sleep disorders do not only affect health, but may also influence work quality and productivity. Therefore, it is crucial to promote sleep quality and develop targeted prevention and intervention measures.

Inflammatory cytokines are active signaling molecules secreted by immune cells. In addition to participating in the immune response, Il-6 and TNF-α can also affect neurotransmitter metabolism, neuroplasticity, and neuroendocrine function. They are also related to depression, sleep disorders, and cognitive development (48–53). Previous study has found that the sleep-wake cycle is related to the immune system (22). The results of this study showed that in the analysis of the relationship between sleep quality and cytokines, the IL-1β content was statistically significantly different among different sleep quality groups, suggesting that cytokines may be involved in the sleep-wake process, which is similar to the study by Ren et al. (21). The psychoneurologic-immune model suggested that psychological factors could affect health through immune downregulation. When occupational stress was high, it may regulate the sleep process through immune activation. Cytokines are known to induce other adaptive changes in the central nervous system function, such as activation of the thalamic-pituitary-adrenal (HPA axis) system and, in particular, IL-1β and TNF-α both increase non-rapid eye movement sleep (NREMS). Animal studies have also shown that IL-1β and TNF-α were important mediators of the increase in NREMS volume and intensity (53). Sleep influences two primary effector systems, the HPA axis, and sympathetic nervous system, which together shift the basal gene expression profile toward increased pro-inflammatory type (54, 55). Activation of β-adrenergic signaling induces increase in NF-κB, inflammatory gene expression, production of pro-inflammatory cytokines and markers of systemic inflammatory markers (56). Because sleep is associated with a drop in sympathetic outflow (57), sympathetic effector pathway activation is one such biologically plausible mechanism to explain the associations between sleep quality, short sleep duration, and increases in inflammation markers.

Modern medicine describes the occurrence of insomnia using three major factors: susceptible, inducing, and persistent factors (58). Genetic influence on sleep was first reported in the 1930s, demonstrating greater consistency in sleep parameters between identical and fraternal twins. Genome-wide association studies have shown that some genes and their polymorphisms are associated with sleep problems (59–61). Serotonin (5-HT) is a monoamine neurotransmitter that plays an important role in regulating physiological functions, such as pain and cognition in sleep-eating sexual behavior and temperature (62). Previous studies have shown that of all 5-HT receptor subtypes, 5-HT2A and 5-HT1A receptors are primarily involved in the quantitative and qualitative aspects of wakefulness, NREMS, and REMS (62, 63). This study analyzed the influence of the 5-HT2A receptor gene on sleep, showing that the −1438G/A of *5-HTR2A* gene was associated with sleep quality in non-manual workers and that both AG and GG genotypes could increase the risk of sleep quality problems, which was consistent with the research results by Gao et al. (61). However, the T102C was not associated with sleep quality. A meta-analysis evaluating the association between *5-HTR2A* polymorphism and obstructive sleep apnea (OSA) syndrome found that T102/C of *5-HTR2A* was not a factor in OSA (64). Multivariate logistic regression analysis showed that −1438G/A and IL-1β were both factors affecting sleep quality.

To further explore the relationship among these three factors, the effect of the *5-HTR2A* gene and cytokine interaction on sleep was analyzed. The results showed that the interaction between −1438G/A polymorphism and IL-1β had an effect on sleep quality and served as a protective factor for it, indicating that genetic and physiological factors have a cumulative effect on sleep quality. Simultaneously, this study also suggests that the interaction between *5-HTR2A* gene and cytokines may affect the sleep quality of non-manual workers. Evidence from animal studies has also shown that interactions between immune signaling molecules (such as the cytokine IL-1β) and brain neurochemical systems (such as the serotonin system) are amplified during infection, indicating that these interactions might underlie the changes in sleep that occur during infection (18). IL-1β and 5-HT systems engage in reciprocal interactions that contribute to the regulation of NREM sleep (18).

The present research adopted a cross-sectional study method to explore the relationship among cytokines, *5-HTR2A* polymorphisms, and sleep quality. Both cytokines and *5-HTR2A* polymorphisms were found to affect sleep quality of non-manual workers. A potential interaction between the two factors in the occurrence of sleep quality problems was also identified. That is, the cumulative effect between 5-HTR2A and cytokines was greater than the independent effect. These results may suggest that the 5-HTR2A gene polymorphism may increase the risk of poor sleep quality, but this effect may not be the same in individuals with different IL-1β concentrations.

However, there are still some deficiencies in the present research, and further improvement is needed in future studies. First, the evaluation of sleep quality was based completely on PSQI, a subjective questionnaire survey lacking objective sleep quality evaluation tools and resulting in lower study accuracy. Ideally, laboratory-based polysomnography recordings could be obtained from subjects to confirm the current findings; however, this requires a amount of labor and time, and is also expensive. Second, some confounding factors related to sleep quality, such as shift work, shift frequency, occupational stress and other work-related factors, were not considered in this study. Third, the present study examined the *5-HTR2A* gene polymorphism, but the results would have been more reliable if we had quantified the 5-HT levels of the study participants and examined the relationship between cytokines, 5-HT, *5-HTR2A* gene polymorphisms and sleep quality.

## Conclusion

Both cytokines and *5-HTR2A* polymorphisms are associated with sleep quality, and both can be used as predictors of sleep quality problems. In addition, there may be an interaction between cytokines and *5-HTR2A* polymorphisms when considering sleep quality. Specifically, effective detection of IL-1β can influence the independent effect of *5-HTR2A* polymorphisms on sleep quality. Future research will require a larger sample size and a cohort study to verify the association among these factors and provide a stronger theoretical basis for improving sleep quality in non-manual workers.

## Data Availability Statement

The original contributions presented in the study are included in the article/supplementary materials, further inquiries can be directed to the corresponding author/s.

## Ethics Statement

The studies involving human participants were reviewed and approved by the Ethics Committee of Xinjiang Medical University. The patients/participants provided their written informed consent to participate in this study.

## Author Contributions

JW, XG, PG, and JL designed the study, contributed to the acquisition, analysis, interpretation of data, involved in drafting the manuscript, and revising it for important intellectual content. All authors contributed substantially to the work presented in this paper, reviewed, and approved the final manuscript.

## Funding

This study was funded by the National Natural Science Foundation of China (Grant No. 81760581).

## Conflict of Interest

The authors declare that the research was conducted in the absence of any commercial or financial relationships that could be construed as a potential conflict of interest.

## Publisher's Note

All claims expressed in this article are solely those of the authors and do not necessarily represent those of their affiliated organizations, or those of the publisher, the editors and the reviewers. Any product that may be evaluated in this article, or claim that may be made by its manufacturer, is not guaranteed or endorsed by the publisher.
